# Energy Charge as an Indicator of Pexophagy in *Pichia pastoris*

**DOI:** 10.3389/fmicb.2017.00963

**Published:** 2017-05-30

**Authors:** Jianguo Zhang, Taiyu Liu

**Affiliations:** Institute of Food Science and Engineering, University of Shanghai for Science and TechnologyShanghai, China

**Keywords:** *Pichia pastoris*, pexophagy, energy charge, alcohol oxidase, formate dehydrogenase

## Abstract

*Pichia pastoris* is a good model for pexophagy research owing to its diverse pexophagy modes (macropexophagy and micropexophagy) exhibited during carbon-source shift from methanol to other carbon sources. The critical condition that triggers activation of macropexophagy and micropexophagy is important for clarifying the *P. pastoris* pexophagy mechanism and human peroxisomal disorders. In this study, the pexophagy modes of *P. pastoris* were confirmed by green fluorescent protein expression and alcohol oxidase and formate dehydrogenase activities. Furthermore, intracellular energy charge (EC) was found to be a determinant of pexophagy activation. During methanol induction, the EC was about 0.5. And the final EC value was related to the pexophagy mode when carbon source switched from methanol to others. Macropexophagy and micropexophagy occurred when the EC increased to 0.6–0.75 and above 0.75, respectively. Thus, different EC values were considered as the important factor to trigger different pexophagy modes in *P. pastoris*. The results obtained in this study could help in achieving better control of the pexophagy modes to study the pexophagy mechanism.

## Introduction

Peroxisomes are ubiquitous and dynamic organelles involved in diverse metabolic reactions, such as oxidation of long chain fatty acid and amino acids, reduction of reactive oxygen species in response to environmental changes in eukaryotic organisms. Pexophagy or peroxisome autophagy, named for a selective degradation pathway of peroxisomes, is a kind of self-regulation pattern exhibited by cells for waste cleaning and resource recycling (Islinger et al., 2012; Sibirny, 2016). Pexophagy was described in many organelles of mammalian cell, plant, fungi, yeasts (Manjithaya et al., 2010). According to Nazarko’s (2017) report, pexophagy is responsible for 65% cases of peroxisome biogenesis disorders. The clarification of the pexophagy mechanism help in better understanding of the cellular protection pathway under hostile conditions, as well as benefit treatments of human peroxisomal disorders such as Zellweger syndrome, neonatal adrenoleukodystrophy, infantile Refsum’s disease, and Rhizomelic chondrodysplasia punctata (Steinberg et al., 2006). It is interested that similar genetic defects are observed in methylotrophic yeasts, such as *Hansenula polymorpha* and *Pichia pastoris*. *P. pastoris* is an attractive model for pexophagy research owing to the following reasons. First, *P. pastoris* exhibits two distinct modes of pexophagy (macropexophagy and micropexophagy) during carbon-source shift from methanol to other carbon sources (Tuttle and Dunn, 1995). Macropexophagy occurs when the carbon source is switched from methanol to ethanol. In this mode, peroxisome is selectively sequenced by a newly synthesized membrane termed pexophagosome which then fuses with the vacuole and is degraded by proteinase in the vacuole. Micropexophagy occurs when the carbon source is switched from methanol to glucose or glycerol. In this mode, vacuolar forms protrusions along with a clusters of peroxisomes. A double-membrane, the micropexophagy-specific membrane apparatus (MIPA), is synthesized at the peroxisome surface when peroxisomes is nearly enclosed. Finally, peroxisomes are engulfed into a vacuole and then degraded by proteinase in the vacuole. Both these modes are complicated processes involving over 30 proteins named as Atg (autophagy-related proteins). The number and sequence of Atg involved in these two pexophagy processes have been widely studied (Sibirny, 2014). Second, when *P. pastoris* grows in media containing methanol as the sole carbon source, the peroxisomes rapidly proliferate, reaching up to 40% of the cell volume. These large peroxisome clusters facilitate fluorescence imaging and biochemical analysis of peroxisomes. Lastly, the process of pexophagy in *P. pastoris* is rapid, which saves time and facilitates studies on pexophagy (De Schutter et al., 2009).

For *P. pastoris* pexophagy, receptor Atg30 phosphorylation is a prerequisite for its interactions with the autophagy scaffold proteins, Atg11 and Atg8. Subsequently, Atg30 is shuttled to the vacuole along with the targeted peroxisome for degradation. However, the sensing and signaling of pexophagy need to be further investigated to understand the complete route (Sibirny, 2014). Thus, a stable condition to trigger the activation of pexophagy is important for *P. pastoris* research. It has been previously claimed that intracellular ATP level is a key factor determining the modes of pexophagy (Ano et al., 2005). This relationship was also described by other researchers (Yuan et al., 1997; Till et al., 2012). In the present study, energy charge (EC) was noted to be a vital factor controlling the modes of pexophagy.

In addition, *P. pastoris* is a powerful producer of heterologous proteins because of its high-level heterologous protein production, strict gene expression mechanism of alcohol oxidase 1 (AOX1) promoter, easy cultivation, and high cell-density fermentation (Potvin et al., 2012). Heterologous gene under AOX1 promoter is induced strongly by methanol because of its low affinity of AOX for oxygen, and inhibited by glucose, glycerol, or ethanol. The modification of glycosylation pathway in *P. pastoris* has enabled its application in the industrial production of therapeutic proteins (Hamilton et al., 2003). The heterologous proteins produced from *P. pastoris* have been approved by the FDA and are available in the market (Vogl et al., 2013). However, low energy provided is a drawback for higher heterologous protein expression because of low methanol metabolism rate of *P. pastoris* (Looser et al., 2015). Methanol mixed with glycerol or glucose is used to maximize the energy metabolism for high heterologous protein expression because of high metabolite rate of glycerol or glucose of *P. pastoris*. At this condition, partial peroxisomal metabolism is no longer required. The peroxisomes become redundant and are subject to degradation by the vacuole through pexophagy pathway. Therefore, heterologous gene under AOX1 promoter does not express highly (Zhang et al., 2010). Therefore, an understanding of the pexophagy mechanism could be also beneficial for the heterologous protein production in *P. pastoris*.

## Materials and Methods

The *E. coli* strain JM109 served as plasmid host. Standard procedures were used for all recombinant DNA manipulations (Green and Sambrook, 2012). The *P. pastoris* GS115 cells were obtained from Life Technologies (Carlsbad, CA, United States). The biochemicals were purchased from Takara (Takara, Dalian, China), and the chemicals were purchased from Sino Chemicals (Shanghai, China). The media employed in the present study were as follows: yeast extract peptone dextrose (YPD) medium (containing 10 g/L yeast extract, 20 g/L peptone, and 20 g/L glucose; for YPD agar plates, 15 g/L agar was also included); lysogeny broth (LB) medium (containing 5 g/L yeast extract, 10 g/L peptone, and 10 g/L NaCl; pH was adjusted to 7.0 using 1 mmol/L NaOH); low salt lysogeny broth (LLB) medium (containing 5 g/L yeast extract, 10 g/L peptone, and 5 g/L NaCl; pH was adjusted to 7.0 using 1 mmol/L NaOH); super optimal broth with catabolite repression (SOC) medium (containing 0.5 g/L yeast extract, 2 g/L peptone, 0.05 g/L NaCl, 2.5 mmol/L KCl, 10 mmol/L MgCl_2_, and 20 mmol/L glucose); and minimal methanol medium [1.34% yeast nitrogen base (YNB, *Pichia* Expression Kit, Invitrogen, Carlsbad, CA, United States), 4 × 10^-5^% biotin, 0.5% methanol].

### Green Fluorescent Protein Expression in Recombinant *P. pastoris* Cells

The green fluorescent protein (GFP) gene (GFPN1) was obtained by amplification using primers (forward: 5′-TCGGTACCTCGAGCCGCGGCCATGGTGAGCAAGGGCGAG-3′, reverse: 5′-AAGCTGGCGGCCGCCGCGGTTATAATTTGGACTTGTACAGCTCGTCC-3′) and the vector pEGFPN1 (4.7 kb) as template. The amplified sequence contained serine-lysine-leucine (SKL, TTATAATTTGG) and a 15-bp homologous arm. The SKL sequence is the consensus peroxisomal targeting sequence 1 (PTS1), which was noted to direct GFP to peroxisomes *in vivo*. The 15-bp homologous arm was employed to ligate GFPN1 and digested pPICZA using the In-Fusion HD Cloning Kit (Takara, Dalian, China) to construct the vector pPICZA-GFP. The vector pPICZA-GFP was transferred into *P. pastoris* GS115 by electrotransformation. All the methods were performed according to the *Pichia* protocol (Life Technologies, Carlsbad, CA, United States). The recombinant *P. pastoris* cells were cultivated in YPD medium with 100 μg/mL zeocin at 30°C and 180 rpm for 3 days. Subsequently, a single colony was inoculated into a 20-mL tube containing 5 mL of YPD medium and incubated at 30°C and 180 rpm for 18–20 h. Then, the YPD broth was transferred into a 250-mL flask containing 50 mL of MM medium and incubated at 30°C and 180 rpm for 24 h. The recombinant *P. pastoris* cells were induced by adding 0.5% methanol every 24 h for 72 h. For pexophagy process, different concentration of glucose or ethanol was added after 72 h methanol induction.

### FM4-64 Fluorescence Detection

This method was carried out according to the protocol of monitoring autophagy in yeast using FM 4-64 fluorescence (Journo et al., 2008). The dye FM4-64 was dissolved in dimethyl sulfoxide (DMSO) to obtain an 8 mmol/L FM4-64 solution. After 48 h of induction, the *P. pastoris* cells were harvested by centrifugation (3000 rpm and 5 min) and suspended in MM medium to obtain cell broth (OD_600_ of about 8.0). Then, 5 μL of the FM4-64 solution were added to 5 mL of the cell broth and incubated at 30°C for 30 min in a tube covered with silver paper. Subsequently, the mixture was washed with 5 mL of 10 mmol/L phosphate-buffered saline (PBS, pH 7.5), re-suspended in 5 mL of MM medium, and incubated in a shaker at 30°C and 180 rpm for 30 min. The *P. pastoris* cells were detected using fluorescence microscopy (DM IL LED, Leica, Wetzlar, Germany) according to the manufacturer’s instructions.

### Determination of Alcohol Oxidase and Formate Dehydrogenase Activities

The enzyme extracts were obtained from *P. pastoris* cells by using glass beads as reported earlier (Tam et al., 2012). AOX activity was determined using methanol as the substrate, which produced hydrogen peroxide. The enzyme extract was incubated with 10 U/mL horseradish peroxidase, 10 mmol/L PBS (pH 7.5), 1 mmol/L 4-aminoantipyrine, 4.3 mol/L phenol, and 200 mmol/L methanol at 37°C for 10 min. A red-colored product was obtained, which was evaluated using a spectrophotometer at 500 nm (722S, Shanghai Precision Scientific Instrument Co., Ltd, Shanghai, China) with the modification of Schroder’s method (Schroder et al., 2007). One unit of AOX activity was defined as the amount of AOX required to produce 1 μmol H_2_O_2_ per minute.

Formate dehydrogenase (FDH) activity was assayed using sodium formate as the substrate, along with reduction of NAD^+^ at 340 nm, according to the procedure developed by Kato (1990) with modification. The *P. pastoris* lysis solution was mixed with 1.62 mmol/L NAD^+^, 162 mmol/L sodium formate, and 100 mmol/L PBS (pH 7.5), and incubated in a water bath at 30°C for 10 min. Subsequently, the absorbance was measured at OD_340_ using a spectrophotometer (722S, Shanghai Precision Scientific Instrument Co., Ltd, Shanghai, China). One unit of FDH activity was defined as the amount of FDH required to produce 1 μmol NADH per minute.

Protein concentration was determined by the method of Bradford (1976). AOX and FDH activities (U/mg) were used to represent the activities in *P. pastoris* cells.

### Determination of ATP, ADP, and AMP Concentrations and EC Calculation

After washing with deionized water, the *P. pastoris* cells (2 mL) were suspended in 0.5 mL of deionized water and 0.5 mL of 20% perchloric acid for 10 min. The pH of the suspension was adjusted to 3.0 using 1 mL of 1.3 mol/L KOH. Subsequently, the suspension was centrifuged (10,000 rpm, 1 min) and filtered through a 0.22-μm membrane to remove potassium perchlorate, and the pH was further adjusted to 7.0 using 1 mL of 100 mmol/L PBS (pH 7.5). The obtained solution was stored at -20°C until further analysis. High performance liquid chromatography (Waters 2480, MA, United States) equipped with a reverse phase C18 column (4.6 mm × 250 mm, 5 μm) was performed with the stored solution and 100 mol/L PBS (pH 6.25) at a flow rate of 1.0 mL/min. The ATP, ADP, and AMP concentrations were calculated based on the peak areas at 254 nm, and EC was calculated using Eq. 1.

(1) $$\text{EC}=\frac{\text{ATP}+0.5\text{ADP}}{\text{ATP}+\text{ADP}+\text{AMP}}.$$

### Data Statistical Analysis

All experiments were carried three times. The statistical analysis was performed by using Microsoft Excel 2016. The standard deviation was calculated which was showed as error bar. One-way Student’s *t*-test was chosen for the treatment comparisons for difference. All used statistics were based on a confidence level of 95%, hence *p* values smaller than 0.05 were considered statistical significance.

## Results

### GFP Expression in *P. pastoris* Cells for Peroxisome Localization and Pexophagy Visualization

**FIGURE 1:**
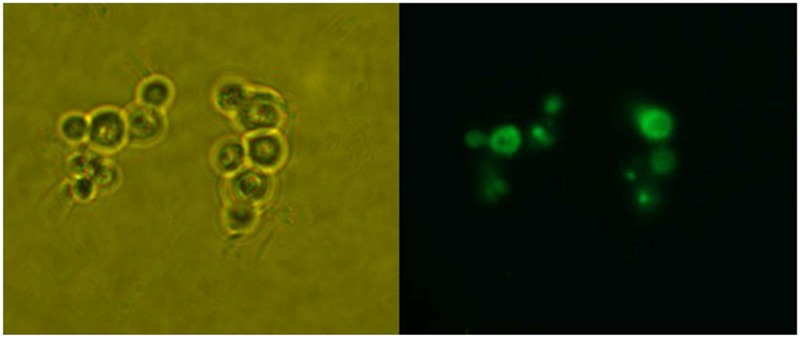
Detection of GFP expression in *P. pastoris*. Left: *P. pastoris* cells under microscope; Right: *P. pastoris* cells under fluorescence microscope.

**FIGURE 2:**
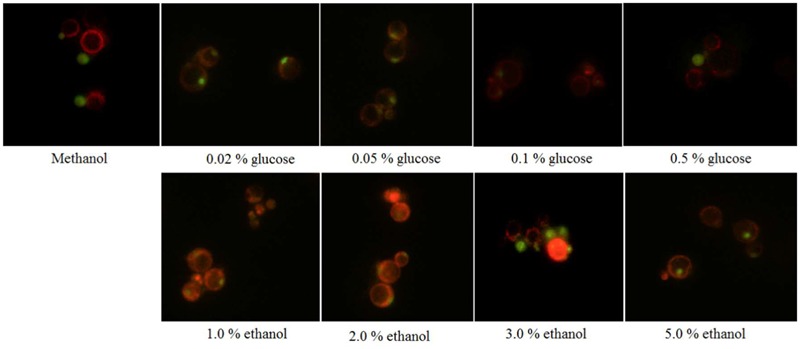
*Pichia pastoris* cells when the carbon source was switched from methanol to glucose or ethanol.

The restriction enzyme *Sac*I was used to digest the recombinant vector pPICZA-GFPN1 to obtain the linearized vector for homologous recombinant with *P. pastoris*. The fluorescence results are shown in **Figure 1**. The *P. pastoris* cells appeared green under fluorescence microscope, which indicated that the peroxisomes were labeled with GFP. Furthermore, the *P. pastoris* vacuole was labeled with FM4-64, which appeared red under fluorescence microscope. **Figure 2** shows the recombinant *P. pastoris* cells after 2 h of incubation in non-methanol carbon sources. The peroxisomes were engulfed by the vacuole during incubation in ethanol or glucose. On the one hand, when the carbon source was changed from 0.5% methanol to 0.02% glucose, 0.05% glucose, 1.0% ethanol, and 2.0% ethanol, respectively, no significant changes in the shapes of the vacuoles were observed. On the other hand, when the carbon source was switched from 0.5% methanol to 0.1% glucose, 0.5% glucose, 3.0% ethanol, and 5.0% ethanol, respectively, significant changes in the shapes of the vacuoles were noted.

### AOX and FDH Activities during Carbon-Source Switch

**FIGURE 3:**
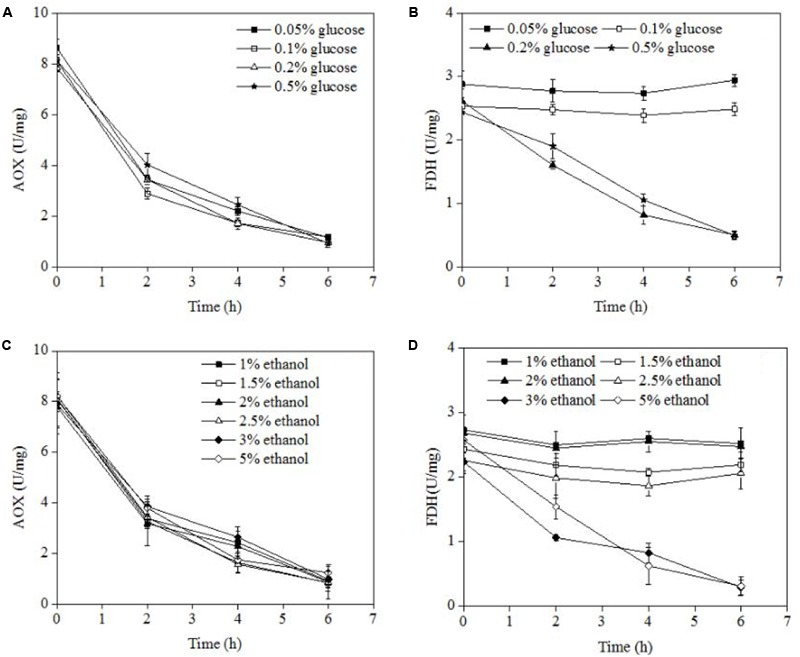
AOX and FDH activities in *P. pastoris* cells at different concentrations of various carbon sources. **(A)** AOX activities of *P. pastoris* cells when carbon source was switched from 0.5% methanol to different concentrations of glucose. **(B)** FDH activities of *P. pastoris* cells when carbon source was switched from 0.5% methanol to different concentrations of glucose. **(C)** AOX activities of P. pastoris cells when carbon source was switched from 0.5% methanol to different concentrations of ethanol. **(D)** AOX activities of *P. pastoris* cells when carbon source was switched from 0.5% methanol to different concentrations of ethanol.

AOX and FDH were two major enzymes for methanol oxidase. AOX was degraded during pexophagy because AOX stated in peroxisomes. For FDH was degraded only during micropexophagy, not during macropexophagy. The AOX and FDH activities were measured at different concentrations of glucose or ethanol after methanol induction, and the results are shown in **Figure 3**. At all the concentrations of glucose and ethanol examined, the AOX activity decreased from about 8 to 0.5 U/mg after 6 h of incubation. In the case of FDH, a decrease in its activity was noted at a glucose concentration of >0.2% or an ethanol concentration of >3.0%. However, the FDH activity remained constant when the glucose concentration was <0.2% or ethanol concentration was <3.0%. Therefore, it can be concluded that micropexophagy occurred at a glucose concentration of >0.2% or an ethanol concentration of >3.0%, whereas macropexophagy occurred at a glucose concentration of <0.2% or an ethanol concentration of <3.0%.

### ATP, ADP, AMP, EC Profile during Pexophagy

**FIGURE 4:**
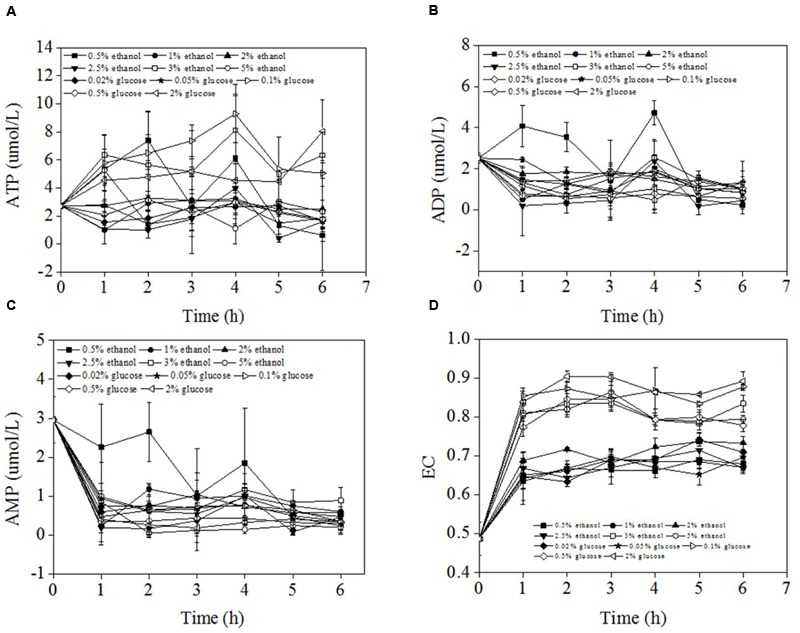
ATP, ADP, AMP concentrations, and EC of *P. pastoris* cells when the carbon source was switched from 0.5% methanol to other carbon sources. **(A)** ATP conncentrations of *P. pastoris* during carbon source shifting from 0.5% methanol to different concentrations of ethanol or glucose. **(B)** ADP conncentrations of *P. pastoris* during carbon source shifting from 0.5% methanol to different concentrations of ethanol or glucose. **(C)** AMP conncentrations of *P. pastoris* during carbon source shifting from 0.5% methanol to different concentrations of ethanol or glucose. **(D)** EC values of P. pastorisd during carbon source shifting from 0.5% methanol to different concentrations of ethanol or glucose.

**Figure 4** showed the ATP, ADP, AMP concentrations, and EC values during carbon source shifting from 0.5% methanol to different concentrations of ethanol or glucose. The overall ATP concentrations fluctuated between 1 and 8 μmol/L. There was no significant gap between ATP concentrations of different pexophagy modes although there was a tendency of high ATP concentrations at high carbon source concentrations. The ADP concentrations fluctuated between 0 and 4 μmol/L with a trend of decreasing slightly. AMP concentrations decreased from 3 μmol/L to about 0.5 μmol/L within 1 h of carbon switch except that of 0.5% ethanol condition. After 1 h carbon source switch, the AMP concentrations kept stable. While AMP concentration of 0.5% ethanol decreased gradually within 5 h of carbon switch. The ADP, AMP concentrations of different pexophagy modes also did not show difference as showed in **Figures 4B,C**. The ECs of *P. pastoris* cells were determined within 6 h after the carbon source was switched from 0.5% methanol to different concentrations of several other carbon sources (**Figure 4D**). The results revealed that the ECs of *P. pastoris* cells significantly increased from 0.48 (in the presence of 0.5% methanol) to 0.6–0.9. The *p* value between EC values of micropexophagy and macropexophagy was 1.89 × 10^-22^ which is significantly different. The final EC varied according to different concentrations of carbon sources. High ECs were noted when high concentrations of ethanol or glucose were used to replace 0.5% methanol. During micropexophagy, which was noted when *P. pastoris* was cultivated at a glucose concentration of >0.2% or ethanol concentration of >3.0%, the final ECs were >0.75. In contrast, during macropexophagy, which was observed when *P. pastoris* was cultivated at a glucose concentration of <0.2% or ethanol concentration of <3.0%, the final ECs were <0.75. Therefore, the EC values of two pexophagy models were different, which were considered as indicator for the divergent pexophagy modes.

## Discussion

The metabolism rate of methanol, glucose, ethanol of *P. pastoris* was effected by the cultivation scale, and cultivation process. The maximal specific growth rate of *P. pastoris* varied from 0.093 to 0.28 h^-1^ (Looser et al., 2015). The pexophagy occurred at the non-methanol carbon source used. All the AOX, FDH, and peroxisome morphology were necessary to verify the pexophagy of *P. pastoris*. This research used the GFP labeled peroxisome for pexophagy observation (**Figure 2**). And AOX and FDH activities were determined at the same time to confirm the micropexophagy and macropexophagy.

In the present study, the FDH activity did not significantly decrease when the glucose concentration was <0.05% or ethanol concentration was <2.5%. However, the AOX activity significantly decreased regardless of the type or concentration of carbon sources. While it was reported that the different modes of *P. pastoris* pexophagy were recognized by the changes in the FDH activity following switching of the carbon source from methanol to other carbon sources (Tuttle and Dunn, 1995). The results obtained in the present study indicated that different modes of pexophagy occurred at different concentrations of carbon sources. As the critical concentration of various carbon sources differed, the EC was analyzed as a crucial parameter to induce different modes of pexophagy. A high EC value for micropexophagy suggested the need for high energy for protein synthesis in this mode as well as the energy-demanding property of vacuolar dynamics. This finding was constant to results of micropexophagy inhibition with the addition of cycloheximide (Sakai et al., 1998) which is an inhibitor of protein synthesis, and wortmannin. However, macropexophagy was not inhibited by cycloheximide addition. Wortmannin was a specific inhibitor of PI3-kinase and inhibited the micropexophagy at the stage of vacuole invagination to peroxisome (Stack and Emr, 1994). As atg30 was a key player in the selection of peroxisomes. And the increasing of atg30 phosphorylation was required for pexophagy. Low level (5–10%) of atg30 phosphorylation in *P. pastoris* growing on methanol increased to 80–90% proportion of atg30 phosphorylation. This could be the reason of high EC at the condition of glucose or ethanol. As more atgs were needed for pexophagy, it could be concluded that micropexophagy requires more energy to synthesize new proteins, and hence occurs under high EC condition. The EC value determined in the present study has potential applications in the analysis of the mechanisms of the two pexophagy modes. In particular, the EC value could act as an indicator of the pexophagy modes and help in better understanding of the pexophagy mechanism, not only in *P. pastoris*, but also in other yeasts (Oku and Sakai, 2016).

## Author Contributions

JZ provides the conception, designs the work, helps TL to get and analyze data, writes the manuscript. TL does experiments, drafts the manuscript, revises it critically. Both authors approval of the version to be published; and agree to be accountable for all aspects of the work.

## Conflict of Interest Statement

The authors declare that the research was conducted in the absence of any commercial or financial relationships that could be construed as a potential conflict of interest.
